# Comprehensive analysis of morbidity and mortality patterns in familial partial lipodystrophy patients: insights from a population study

**DOI:** 10.3389/fendo.2024.1359211

**Published:** 2024-06-03

**Authors:** Natália Rossin Guidorizzi, Cynthia M. Valerio, Luiz F. Viola, Victor Rezende Veras, Virgínia Oliveira Fernandes, Grayce Ellen da Cruz Paiva Lima, Amanda Caboclo Flor, Jessica Silveira Araújo, Raquel Beatriz Gonçalves Muniz, Rodrigo Oliveira Moreira, Francisco José Albuquerque De Paula, Lenita Zajdenverg, Joana R. Dantas, Amélio F. Godoy-Matos, Renan Magalhães Montenegro Júnior, Maria Cristina Foss-Freitas

**Affiliations:** ^1^ Brazilian Group for the Study of Inherited and Acquired Lipodystrophies (BRAZLIPO), Fortaleza, Brazil; ^2^ Division of Endocrinology and Metabology, Department of Internal Medicine, Clinical Hospital of Ribeirão Preto Medicine School, University of São Paulo, Ribeirão Preto, Brazil; ^3^ Department of Metabolism, Institute of Diabetes and Endocrinology of Rio de Janeiro (IEDE), Rio de Janeiro, Brazil; ^4^ Rondonópolis Diabetes and Endocrinology Center (CEDERO), Rondonópolis, Brazil; ^5^ Clinical Research Unit, Walter Cantídio University Hospital, Federal University of Ceará/Empresa Brasileira de Serviços Hospitalares - Brazilian Hospital Services Company (EBSERH), Fortaleza, Brazil; ^6^ Internal Medicine Department - Nutrology and Diabetes Session, Federal University of Rio de Janeiro (UFRJ), Rio de Janeiro, Brazil; ^7^ Division of Metabolism, Endocrinology and Diabetes (MEND), Department of Internal Medicine, Michigan Medicine, University of Michigan, Ann Arbor, MI, United States

**Keywords:** familial partial lipodystrophy, morbidity and mortality, insulin resistance, diabetes *mellitus*, hypertriglyceridemia, cardiovascular disease

## Abstract

**Background:**

There is a lack of information on the clinical and molecular presentation of familial partial lipodystrophy (FPLD), a rare genetic disorder characterized by partial subcutaneous fat loss.

**Objective:**

This study aimed to provide a comprehensive assessment of the clinical, metabolic, and genetic features of FPLD in the Brazilian population.

**Methods:**

In a multicenter cross-sectional investigation we evaluated patients with FPLD across five Brazilian reference centers for lipodystrophies. Diagnosis of FPLD was made by clinical evaluation and genetic confirmation. Data on genetic, clinical, and metabolic characteristics were captured. Statistical analysis involved the utilization of the Kruskal-Wallis test to identify differences.

**Results:**

The study included 106 patients with genetic confirmation of FPLD. The mean age was 44 ± 15 years, and they were predominantly female (78.3%). *LMNA* pathogenic variants were identified in 85.8% of patients, *PPARG* in 10.4%, *PLIN1* in 2.8%, and *MFN2* in 0.9%. Diabetes *mellitus* (DM) was highly prevalent (57.5%), affecting 54 females (50.9%). Median triglycerides levels were 199 mg/dL (54–2724 mg/dL), severe hypertriglyceridemia (≥ 500 mg/dL) was found in 34.9% and pancreatitis in 8.5%. Metabolic-associated fatty liver disease (MAFLD) was observed in 56.6%, and cardiovascular disease in 10.4%. The overall mortality rate was 3.8%, due to cardiovascular events.

**Conclusion:**

This study presents an extensive cohort of Brazilian patients with FPLD, predominantly DM with several multisystem complications. A comprehensive characterization of lipodystrophy syndromes is crucial for effective patient management and care.

## Introduction

Partial lipodystrophies (PL) represent a rare and diverse group of medical conditions that can arise from genetic or acquired causes, resulting in the partial loss of adipose tissue (AT). They encompass various subtypes based on genetic or clinical factors. The genetic forms of PL, known as familial partial lipodystrophies (FPLD), typically follow autosomal dominant inheritance, except for the Köbberling type (type 1), which lacks an identified genetic mutation. There are seven distinct forms of FPLD, each associated with a specific genetic mutation except for type 1. The various subtypes of FPLD are primarily characterized by the selective loss of subcutaneous AT in the upper and lower limbs, extending to the gluteal region, with variable fat accumulation in the face, neck, and intra-abdominal region. Fat redistribution is a hallmark feature of the condition, typically becoming apparent during late childhood or shortly after puberty (1–5).

The absence of sufficient AT leads to the accumulation of lipids in ectopic tissues and organs such as muscle, liver, pancreas, and epicardial tissue, contributing to insulin resistance and associated complications, including diabetes mellitus (DM), hypertriglyceridemia, polycystic ovary syndrome (PCOS), pancreatitis, metabolic fatty liver disease (MAFLD), and cardiovascular disease (CVD) (2, 6–9).

FPLD often goes underdiagnosed, leading to increased morbidity due to advanced multi-organ complications at the time of late diagnosis. There is an urgent need for a better understanding of the challenges and burden of this disease. In this study, we assess a cohort of Brazilian patients with FPLD, following their progress for over 30 years. In this study, we comprehensively describe these patients’ clinical, metabolic, and genetic characteristics to aid healthcare providers in developing personalized diagnostic criteria for improved clinical practice (1, 2, 4, 7, 10).

## Methods

This cross-sectional, retrospective, multicenter study included patients with FPLD from five medical centers in Brazil, all members of the Brazilian Group of Studies on Inherited and Acquired Lipodystrophy (Brazlipo), representing three distinct regions of the country. The diagnosis was primarily clinical, based on identifying partial subcutaneous body fat loss, a critical diagnostic criterion determined by measuring skinfold thickness on the anterior thigh with calipers (≤ 10 mm for men, ≤ 22 mm for women) (11–14). Additional indicators, such as acanthosis nigricans, double chin, and supraclavicular fossa filling, were considered. Clinical-metabolic manifestations, including DM, hypertriglyceridemia, and MAFLD, were also taken into account.

Molecular analysis of genes associated with FPLD was performed to identify pathogenic variants contributing to disease development, including variants of uncertain significance (VUS). Family members of confirmed probands were invited to participate.

Upon obtaining informed consent, blood samples and anthropometric measurements were collected from participants in the medical centers. Variables of interest included sex, age (in years), glycated hemoglobin (A1c) levels, serum triglyceride levels, and high-density cholesterol (HDL-c) levels. A comprehensive medical history documented comorbidities. In some cases where only medical records were available, the local Institutional Review Board waived the need for a consent form for data collection.

DM was defined following the American Diabetes Association, International Diabetes Federation (IDF) criteria and/or the use of antidiabetic agents (15, 16). Poor glycemic control was indicated by A1c ≥ 8.0%. Hypertriglyceridemia was diagnosed at triglycerides levels ≥ 150 mg/dL, with severe hypertriglyceridemia ≥ 500 mg/dL. Low HDL-c levels were measured enzymatically as < 50 mg/dL in women and < 40 mg/dL in men. Pancreatitis was identified based on unequivocally diagnosed by examining the medical records, which included the documentation of at least two of the following criteria: amylase and/or lipase levels exceeding three times the upper limit of normal for the specific testing method, imaging studies revealing clear evidence of pancreatitis, or the presence of intense upper abdominal quadrant pain coupled with hypertriglyceridemia. MAFLD was diagnosed based on the presence of fat infiltration hepatic on dedicated imaging studies and or liver biopsy confirming it, rather than just elevated liver enzymes. Established cardiovascular disease encompassed a history of myocardial infarction, angina, heart failure, stroke, or peripheral arterial disease.

The genetic evaluation utilized a consistent panel of candidate genes across all participating centers. DNA was collected via oral swabs, and genomic analysis employed next-generation sequencing (Illumina technology^®^) with target region capture using probes. Bioinformatics protocols aligned and identified variants concerning the GRCh38 version of the human genome. The ExomeDepth program, an R analysis package bioinformatics tool, was employed to identify copy number variations (CNVs). Medical analysis considered the indication of the test and employed gnomAD as a reference for variant allele frequency. In some instances, genetic evaluation was conducted using the Sanger method with PCR amplification of candidate genes’ specific exons. Nomenclature for identified variants followed recommendations from the Human Genome Variation Society (www.hgvs.org). Cases without identifiable pathogenic variants or those showcasing genes not currently associated with lipodystrophy are outside the scope of this article and will be covered in a separate publication.

The study protocol received approval from the Ethics Committee of the Institute of Diabetes and Endocrinology of the Rio de Janeiro State, Brazil, as the central site and subsequently gained approval from other participating sites.

We utilized the R software (version 4.3.1) in our statistical analysis. We summarized continuous variables by calculating their mean and standard deviation. We used the median and range to represent variables that didn’t follow a normal distribution. Categorical variables were presented through absolute frequencies (denoted as “n”) and relative frequencies expressed as percentages (“%”). We employed the Kruskal-Wallis test to thoroughly examine the data’s distribution characteristics concerning the median (a measure of central tendency). We established a significance level of 5% (p ≤ 0.05) to assess the data rigorously. Decisively rejecting the null hypothesis would indicate a statistically significant distinction in at least one of the groups compared to the others. Following this, we conducted a *post-hoc* analysis using Dunn’s test to understand these distinctions’ specific nature further. We applied the Bonferroni correction to the resulting p-values, ensuring stringent control over the familywise error rate. Any p-value that survived this rigorous adjustment, remaining at or below 0.05, was unequivocally deemed indicative of statistically significant differences among the groups under investigation. Ordinal Eta-squared (H) is a statistical metric used to gauge the strength of the association between ordinal-level independent and dependent variables in statistical analyses. It aids in interpreting the practical significance of research findings when working with ordinal data. This measure typically spans from 0 to 1, with higher H values signifying a more robust relationship between the independent and dependent variables. In practical terms, a more considerable H value suggests that changes in the independent variable can account for a more significant portion of the variation in the ordinal dependent variable. Interpreting H values can be contextual, but a general guideline is as follows: H values around 0.01 indicate a small effect, those near 0.06 suggest a medium effect, and values approximately 0.14 or higher point to a substantial impact. It’s essential to note that these thresholds may vary depending on the specific research context and the field of study.

## Results

We assessed 424 patients clinically diagnosed with FPLD from each study site database. Among them, 106 individuals (25%) had a confirmed positive genetic variant. Moreover, 318 cases did not undergo genetic testing due to loss of follow-up, patient death at the time of the study, or the genetic testing did not find any molecular variant that could justify the lipodystrophy phenotype. Details regarding positive and negative cases per center are presented in **Table 1**. Notably, 11% (n=12) of the positive molecular diagnoses were characterized as a pathogenic variant of uncertain significance (VUS).

**Table 1:** Number of cases and genetic data per center.

CENTER	ALL n (%)	POSITIVE n (%)	NEGATIVE n (%)	*LMNA* n (%)	*MFN2* n (%)	*PPARG* n (%)	*PLIN1* n (%)
FMRP	214 (50.5)	42 (39.6)	172 (54.1)	35 (33.0)	0	4 (3.8)	3 (2.8)
IEDE	113 (26.6)	37 (34.9)	76 (23.9)	32 (30.2)	1 (0.9)	4 (3.8)	0
UFC	60 (14.1)	24 (22.6)	36 (11.3)	21 (19.8)	0	3 (2.8)	0
CEDERO	15 (3.5)	3 (2.8)	12 (3.8)	3 (2.8)	0	0	0
UFRJ	22 (5.2)	0	22 (6.9)	0	0	0	0
ALL	424 (100)	106 (100)	318 (100)	91 (85.8)	1 (0.9)	11 (10.4)	3 (2.8)

FMRP, Ribeirão Preto Medicine School; IEDE, Institute of Diabetes and Endocrinology of Rio de Janeiro; UFC, Federal University of Ceara and Brazilian Group for the Study of Inherited and Acquired Lipodystrophies; CEDERO, Rondonópolis Diabetes and Endocrinology Center; UFRJ, Federal University of Rio de Janeiro; LMNA, lamin A/C gene; PPARG, Peroxisome Proliferator Activated Receptor Gamma gene; PLIN1, Perilipin 1 gene; MFN2, mitofusin 2 gene; n, absolute number; %, percentage of total cases.

In this study, we included only the cases with genetic confirmation in our cohort. The mean age was 44 ± 15 years, predominantly female (78.3%). These 106 individuals with positive genetic testing came from 37 Brazilian families registered in the Brazilian Group for the Study of Inherited and Acquired Lipodystrophies (BRAZLIPO) database (https://www.brazlipo.org/brazlipo/subpages/index.php). Pathogenic variants in the *LMNA* gene were identified in 85.8% of individuals, with *PPARG* in 10.4%, *PLIN1* in 2.8%, and *MFN2* in 0.9% (**Table 1**).

DM was diagnosed in 57.5% of patients, primarily affecting 54 females (50.9%), with 34.9% of these patients displaying HbA1c ≥ 8.0%. Median triglycerides levels were 199 mg/dL (54–2724 mg/dL), and severe hypertriglyceridemia (≥ 500 mg/dL) was seen in 34.9% of the patients. However, pancreatitis was found in 8.5% of patients. MAFLD was observed in most patients (56.6%), and established CVD was present in 10.4%. Additionally, the overall mortality rate was 3.8% (n=4), primarily attributed to cardiovascular events, with a median age of 70 years old (67–71y). Interestingly, all four deceased patients in our cohort had *LMNA* gene mutations affecting codon 482 (**Table 2**).

**Table 2:** Demographic, metabolic, and comorbidities overview of patients with FPLD according to the genetic mutation.

		ALL	LMNA		PPARG	PLIN1	MNF2
			482	non-482			
Female n (%)		83 (78.3)	72 (67.9)		8 (7.5)	2 (1.9)	1 (0.9)
			45 (42.4)	27 (25.5)			
Male n (%)		23 (21.7)	19 (17.9)		3 (2.8)	1 (0.9)	–
			8 (7.5)	11 (10.4)			
Age (y) Mean (± SD)		44 (± 15)	44.7 (± 14.7)		33 (± 13.6)	55.3 (± 12.9)	66 (NA)
			46.3 (± 15.1)	42.4 (± 14)			
A1c (%) Median (range)		6.2 (4.7-13.9)	6.05 (4.7-13.9)		6.3 (5.2-12.4)	7.1 (6.5-9.0)	8.0 (8.0)
			6.2 (4.7-12.6)	5.8 (4.8-13.9)			
A1c ≥ 8.0% n (%)		37 (34.9)	28 (26.4)		6 (5.7)	2 (1.9)	1 (0.9)
			19 (17.9)	9 (8.5)			
Triglycerides (mg/dL) Median (range)		199 (54-2724)	179 (54-2525)		217 (131-2724)	305 (165-531)	219 (219)
			200 (87-1488)	157 (54-2525)			
Triglycerides ≥ 500* mg/dL n (%)		37 (34.9)	26 (24.5) ^#^		9 (8.5)	2 (1.9)	(-)
			17 (16.0) ^&^	9 (8.5) ^&^			
HDL-c* (mg/dL) Median (range)	Female	39 (18-93)	39 (18-93) ^#^		29.5 (19-41)	42.5 (41-44)	45 (45)
			39 (21-65) ^&^	37 (18-93)^&^			
	Male	37 (21-69)	37 (29-69)		23 (21-42)	24 (24)	(-)
			36.5 (29-69)	37 (33-46)			
DM n (%)		61 (57.5)	50 (47.2)		8 (7.5)	2 (1.9)	1 (0.9)
			33 (31.1)	17 (16.0)			
Pancreatitis n (%)		9 (8.5)	6 (5.7)		2 (1.9)	1 (0.9)	(-)
			3 (2.8)	3 (2.8)			
CVD n (%)		11 (10.4)	10 (9.4)		0 (0.0)	1 (0.9)	(-)
			7 (6.7)	3 (2.8)			
MAFLD n (%)		60 (56.6)	54 (50.9)		4 (3.8)	2 (1.9)	(-)
			33 (31.1)	21 (19.8)			

n, absolute number; %, percentage of total cases; SD, standard deviation; A1c, glycated hemoglobin (A1c) levels; HDL-c, high-density lipoprotein-cholesterol; (-), no cases identified; DM, diabetes mellitus; CVD, cardiovascular disease; MAFLD, metabolic-associated fatty liver disease; * p<0.05 between groups; ^#^ p<0.05 comparing LMNA and PPARG groups; ^&^ p<0.05 compared with PPARG; !, percentage about subtype.

Among patients with *LMNA* pathogenic variants, 58.2% were associated with codon 482 of the gene. DM was present in 55% of patients with *LMNA* pathogenic variants, while 73% of patients with *PPARG* and 67% of patients with *PLIN1* pathogenic variants also presented with a diagnosis of DM. A single patient (n=1) with the *MNF2* R707W variant also had DM (**Table 2**).

**Figure 1:**
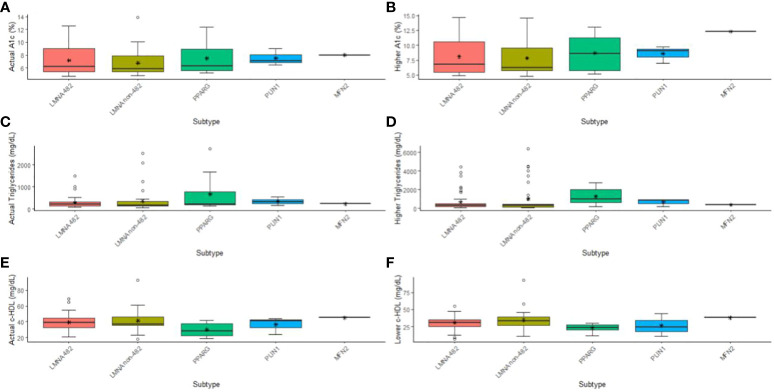
Illustration detailing the affected genes and their subtypes, highlighting the metabolic changes observed in patients, as indicated by median and higher levels of A1c **(A, B)**, and triglycerides **(C, D)**, and median and low levels of HDL-cholesterol **(E, F)**. Patients were divided into two groups based on the location of their LMNA variants: those with mutations occurring at codon 482 and those with mutations occurring at other codons.

In **Figure 1**, we present an illustration detailing the affected genes and their subtypes, highlighting the metabolic changes observed in patients, as indicated by median levels of A1c, HDL-cholesterol, and triglycerides. Notably, patients were divided into two groups based on the location of their *LMNA* variants: those with mutations occurring at codon 482 and those with mutations occurring at other codons. This division was motivated by emerging evidence suggesting that the specific location of *LMNA* variants may correlate with distinct clinical phenotypes and metabolic profiles. By stratifying patients this way, we aimed to explore potential differences in metabolic outcomes and disease progression between these two groups. The key findings from this analysis, which will be discussed further, shed light on the significance of codon-specific *LMNA* mutations in influencing metabolic parameters and clinical outcomes in patients with familial lipodystrophy.

The comparison between the groups of genes showed differences in severe hypertriglyceridemia (p = 0.014), current HDL-c (p = 0.031), and lower HDL-c (p = 0.015) (**Figures 1D–F**). Interestingly, differences were observed between the *LMNA* group and the *PPARG* group regarding severe hypertriglyceridemia (p adj = 0.015), current HDL-c (p adj = 0.028), and HDL-c lowest (p adj = 0.017). No other significant differences were observed among the subtypes. In terms of effect size, as indicated by ordinal Eta-square (H), severe hypertriglyceridemia and lower HDL-c demonstrated a moderate impact.

Upon closer examination of the variants found on the *LMNA* gene, we divided the cohort into those with variants on codon 482 and those on any other codon (482 and non-482, respectively). We found that only severe hypertriglyceridemia (p = 0.030) and lower HDL-c (p = 0.022) had significant differences (**Figures 1D–F**). Moreover, hypertriglyceridemia was significantly higher in the *PPARG* compared to *LMNA* 482 (p adj = 0.049) and non-482 (p adj = 0.033) groups. These differences were associated with a moderate impact (H) in both cases. As for the lowest HDL-c, only the non-482 *LMNA* group displayed a difference from the *PPARG* group (p adj = 0.015), again with a moderate impact (**Figures 1D, F**).

## Discussion

This study contributes significantly to the knowledge of FPLD, illuminating its clinical, genetic, and metabolic complexities. Our pioneering research in Brazil furnishes pivotal insights into the prevalence and attributes of FPLD and accentuates the imperative for early diagnosis and the implementation of comprehensive management strategies.

The prevalence of FPLD has historically been underestimated, with estimates ranging from 1.3 to 4.7 cases per million individuals (14, 17). However, recent research, including the present Brazilian cohort, suggests a higher prevalence, approximately 1 in 20,000 individuals (6, 7, 18). This discrepancy underscores the necessity for heightened awareness and improved diagnostic protocols for this condition. Within the Brazilian cohort, 25% of clinically diagnosed FPLD patients had a confirmatory genetic variant identified. Regarding genetic variants, the *LMNA* gene was the most frequently implicated, affecting 85.8% of individuals, while *PPARG*, *PLIN1*, and variants were less common (1, 6, 7, 10, 17). Among *LMNA* variants, codon 482 was particularly prevalent, and its association with specific metabolic abnormalities was identified. Notably, patients with FPLD3 (associated with *PPARG* pathogenic variants) exhibited a milder lipodystrophy phenotype but a higher prevalence of metabolic complications, such as hypertriglyceridemia and diabetes, emphasizing the clinical heterogeneity within FPLD (1–3). Notably, some of the positive molecular diagnoses were classified as pathogenic VUS, shedding light on the complexity associated with genetic testing in FPLD.

As reported by others, FPLD predominantly affects females, with most male diagnoses arising from cascade screening. This gender disparity may contribute to the underdiagnosis of FPLD in males, and it’s a noteworthy observation emphasizing the importance of cascade screening in this population. A substantial proportion of FPLD patients in this study were diagnosed with DM (57.5%), with a significant majority being female, aligning with existing reports that propose the phenotypic expression of FPLD is more conspicuous in women. Furthermore, it’s noteworthy that approximately one-third of patients with DM presented inadequate glycemic control, as evidenced by A1c levels ≥ 8.0%. Hypertriglyceridemia was a prevalent metabolic abnormality, affecting 34.9% of patients, and severe hypertriglyceridemia was observed in the same proportion. Pancreatitis, a severe complication, while less common, was still present in 8.5% of the patients and underscores the importance of early diagnosis and management. The prevalence of MAFLD was substantial, affecting over half of the patients. Notably, all four patients who died from acute myocardial infarction had pathogenic variants in the *LMNA* gene affecting codon 482. *Akinci et al.* demonstrated an overall survival of 63.9 years, in partial forms of 66.6 years. Our cohort had only four patients with a similar median age. Furthermore, around 33% of the deaths cited were due to cardiovascular disease. We believe that our series is still young, and prospective follow-ups can give us more information about the natural history of the disease. Also, in our sample, most cases had changes in *LMNA* 482, which could be a bias in this data. This observation suggests a potential genetic association between specific *LMNA* variants and cardiovascular complications. It aligns with previous research highlighting the cardiac impact of LMNA mutations in patients with FPLD and emphasizes the need for vigilant cardiac monitoring in these patients. The risk of arrhythmias, atrial fibrillation, and other cardiac events was notably higher in those with *LMNA* variants (1, 6, 7, 19, 20).

The study revealed significant differences among patient groups based on genetic variants and subtypes. *LMNA* codon 482 mutations were associated with specific metabolic features, particularly severe hypertriglyceridemia and lower HDL-c levels. These findings suggest that genetic subtyping can provide valuable insights into the clinical course of FPLD (1, 6).

The association of *LMNA* with various phenotypes, including cardiac disorders and neuromuscular diseases, prompts whether these factors contribute to the clinical metabolic changes observed in lipodystrophy. Mutations in *LMNA* are implicated in a range of conditions known as laminopathies, which exhibit diverse phenotypes encompassing disorders affecting muscles, axonal neurons, progeroid syndromes, and lipodystrophies. While *LMNA* mutations are most commonly reported in patients with familial partial lipodystrophy (FPLD) of the Dunnigan variety, phenotypic heterogeneity exists in the distribution of body fat loss. Familial lipodystrophy, characterized by metabolic anomalies, skeletal muscle deviations, and adipose tissue alterations, can manifest as cardiomyopathy. Clinical reports often describe cardiac manifestations, typically resembling hypertrophic cardiomyopathy, with some cases progressing to left ventricular systolic dysfunction. However, data on the prevalence of cardiac functional abnormalities, such as diastolic dysfunction or early signs of systolic dysfunction, are lacking. The precise pathophysiological mechanisms remain unclear despite the common association between congenital lipodystrophy and ventricular hypertrophy. Speculation exists that disrupted glucose metabolism associated with the condition may activate pro-hypertrophy genes, potentially explaining this correlation. Furthermore, it’s crucial to consider the involvement of laminopathies and the neuromuscular presentation in the intricate interplay between familial lipodystrophy and its cardiac manifestations (7, 8, 20).

Comparing these findings to other international studies, individuals with FPLD in Brazil appear to experience a reduced life expectancy, with cardiovascular disease emerging as the predominant cause of death. This highlights the critical importance of early diagnosis, monitoring, and appropriate management to extend the life expectancy of FPLD patients. Moreover, the study contributes to the growing body of evidence indicating that specific genetic variants, such as *LMNA* mutations, may confer a higher risk of metabolic and cardiac complications (1, 6, 7, 10).

The study’s outcomes bring to the forefront local disparities and potential founder effects in the prevalence of FPLD, particularly evident within specific Brazilian states (21). (21) The existence of distinct genetic mutations within these regions may substantiate the observed higher prevalence rates. Accordingly, it is warranted that further exploration of local environmental and genetic determinants be undertaken to attain a more comprehensive understanding of the epidemiological landscape of FPLD in Brazil.

We acknowledge the limitations of this study. Primarily, the retrospective nature of the investigation, spanning over three decades, introduced complexities tied to temporal variations in data standardization. Furthermore, excluding data from patients without genetic evaluations or negative genetic findings bears the potential to introduce bias. Thus, including negative cases in future research is imperative for comprehensively understanding FPLD in Brazil.

These acknowledged limitations accentuate the need for further studies and collaborative efforts to comprehend FPLD fully. Subsequent research endeavors should focus on standardized data collection, explore plausible regional variations, and delve into the epidemiological intricacies of this often underdiagnosed disease. Our data show for the first time that Brazilian patients with FPLD have multi-system complications, with a high prevalence of DM, hypertriglyceridemia, MAFLD, and CVD. These data highlight the importance of appropriate follow-up of patients with FPLD to prevent complications and decrease morbidities. Cascade tracing has shown to be a valuable tool for identifying the disease early and is responsible for the high number of FPLD patients identified. Finally, this reinforces the efforts to create a registry with clinical, metabolic, and genetic characteristics of FLPD to guide patients’ clinical care and establish appropriate public health policies in Brazil. Our next step is to conduct a national study to determine the prevalence of lipodystrophy.

## Data availability statement

The datasets presented in this study can be found in online repositories. The names of the repository/repositories and accession number(s) can be found in the article/**Supplementary Material** .

## Ethics statement

The studies involving humans were approved by Ethics Committee of the Institute of Diabetes and Endocrinology of the Rio de Janeiro State, Brazil, as the central site, and subsequently gained approval from other participating sites. The studies were conducted in accordance with the local legislation and institutional requirements. The participants provided their written informed consent to participate in this study.

## Author contributions

NG: Conceptualization, Data curation, Formal analysis, Investigation, Methodology, Software, Writing – original draft, Writing – review & editing. CV: Conceptualization, Data curation, Investigation, Supervision, Validation, Visualization, Writing – original draft, Writing – review & editing. RMM: Conceptualization, Data curation, Funding acquisition, Resources, Supervision, Validation, Visualization, Writing – original draft, Writing – review & editing. LV: Conceptualization, Data curation, Investigation, Validation, Visualization, Writing – original draft, Writing – review & editing. VV: Writing – review & editing. VF: Writing – review & editing. GL: Writing – review & editing. AF: Writing – review & editing. JA: Writing – review & editing. RG: Writing – review & editing. ROM: Writing – review & editing. FP: Writing – review & editing. LZ: Writing – review & editing. JD: Writing – review & editing. AG-M: Writing – review & editing. MF-F: Conceptualization, Data curation, Formal analysis, Investigation, Methodology, Project administration, Resources, Supervision, Validation, Visualization, Writing – original draft, Writing – review & editing.

## Appendix

**Table:** APPENDIX 1 Supplementary detailed genetic data.

Number of patients	Affected gene	c-DNA level	Protein level	ACMG classification	Description in the literature*
10	NM_170707.4(*LMNA*)	c.1930C>T	p.Arg644Cys (R644C)	Uncertain significance	March 2023
50	NM_170707.4(*LMNA*)	c.1444C>T	p.Arg482Trp(R482W)	Pathogenic	April 2022
3	NM_170707.4(*LMNA*)	c.1445G>A	p.Arg482Gln (R482Q)	Pathogenic	February 2024
4	NM_170707.4(*LMNA*)	c.1751G>A	p.Arg584His (R584H)	Uncertain significance	December 2021
1	NM_170707.4(*LMNA*)	c.1748C>T	p.Ser583Leu (S583L)	Likely pathogenic	July 2023
18	NM_170707.4(*LMNA*)	c.1744C>T	p.Arg582Cys (R582C)	Likely pathogenic	May 2021
5^#^	NM_170707.4(*LMNA*)	c.1396A>G	p.Asn466Asp (N466D)	Likely pathogenic	August 2017
4	NM_138711.6(*PPARG*)	c.964G>A	p.Glu322Lys (E322K)	Uncertain significance	This variant is absent among approximately 125,000 population-based individuals and has not been previously described in the medical literature.
2	NM_138711.6(*PPARG*)	c.893_894insC -	p.Leu298Profs*41 (L298P)	Likely pathogenic	This variant is absent among approximately 125,000 individuals from a population database, which has not been previously described in the literature.
1	NM_138711.6(*PPARG*)	c.802dup	p.Leu268Profs*41 (L268P)	Likely pathogenic	This variant is absent among approximately 125,000 individuals from a population database, which has not been previously described in the literature.
3	NM_138711.6(*PPARG*)	c.846delC	p.Leu283* (L283*)	Likely pathogenic	This variant is absent among approximately 141,000 individuals in the world population and has not been previously described in the literature.
1	NM_138711.6(*PPARG*)	c.560A>G	p.Glu187Gly(E187G)	Uncertain significance	This variant is absent among approximately 125,000 population-based individuals and has not been previously described in the medical literature.
2	NM_002666.5(*PLIN1*)	c.1136G>A	p.Arg379Lys (R379K)	Uncertain significance	This variant is present in heterozygosity in 2 of approximately 125,000 population bank individuals and has not been previously described in medical literature.
1	NM_002666.5(*PLIN1*)	c.355A>G	p.Thr119Ala (T119A)	Uncertain significance	This variant is missing from around 139,000 individuals from population banks and has not been previously described in the literature.
1	NM_014874.4(*MFN2*)	c.2119C>T	p.Arg707Trp (R707W)	Pathogenic	February 2024

ACMG: American College of Medical Genetics.

*Last description. Source: ClinVar (https://www.ncbi.nlm.nih.gov/clinvar/ - accessed on April 5, 2024).

^#^Two individuals presented, in addition to this variant, also a heterozygous variant in the AKT2 gene (chr19:40.238.018 | G > A | ENST00000392038 | c.782C>T | p.Ser261Leu). This variant is only present in heterozygosity. One of approximately 124 thousand individuals from a population bank and not previously described in the literature, therefore form considered a variant of uncertain significance (VUS).
