# Supplementary figures and images for: Comprehensive analysis of morbidity and mortality patterns in familial partial lipodystrophy patients: insights from a population study
Source: Front Endocrinol (Lausanne). 2024 Jun 3;15:1359211. doi: 10.3389/fendo.2024.1359211 (PMC11180885; doi:10.3389/fendo.2024.1359211)

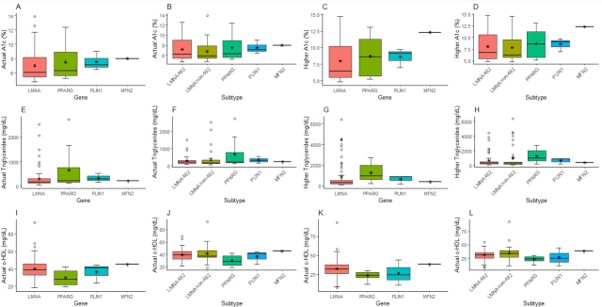

Supplement: Supplementary file 1 [file Image_1.jpeg]
